# Fusion Tensor Subspace Transformation Framework

**DOI:** 10.1371/journal.pone.0066647

**Published:** 2013-07-01

**Authors:** Su-Jing Wang, Chun-Guang Zhou, Xiaolan Fu

**Affiliations:** 1 State Key Laboratory of Brain and Cognitive Science, Institute of Psychology, Chinese Academy of Sciences, Beijing, China; 2 College of Computer Science and Technology, Jilin University, Changchun, Jilin, China; 3 Key Laboratory of Symbolic Computation and Knowledge Engineering of Ministry of Education, Jilin University, Changchun, P.R. China; UC Davis School of Medicine, United States of America

## Abstract

Tensor subspace transformation, a commonly used subspace transformation technique, has gained more and more popularity over the past few years because many objects in the real world can be naturally represented as multidimensional arrays, i.e. tensors. For example, a RGB facial image can be represented as a three-dimensional array (or 3rd-order tensor). The first two dimensionalities (or modes) represent the facial spatial information and the third dimensionality (or mode) represents the color space information. Each mode of the tensor may express a different semantic meaning. Thus different transformation strategies should be applied to different modes of the tensor according to their semantic meanings to obtain the best performance. To the best of our knowledge, there are no existing tensor subspace transformation algorithm which implements different transformation strategies on different modes of a tensor accordingly. In this paper, we propose a fusion tensor subspace transformation framework, a novel idea where different transformation strategies are implemented on separate modes of a tensor. Under the framework, we propose the Fusion Tensor Color Space (FTCS) model for face recognition.

## Introduction

Subspace transformation (or subspace analysis [1]), a main type of feature extraction, has gained huge popularity over the past few years. Principal Component Analysis (PCA) [2] seeks the optimal projection directions according to maximal variances. Linear Discriminant Analysis (LDA) [3] uses discriminant information to search for the directions which are most effective for discrimination by maximizing the ratio between the between-class and within-class scatters. Both PCA and LDA aim to preserve global structures of the samples. Locality Preserving Projections (LPP) [4] aims to preserve the local structure of the original space in the projective subspace. Discriminant Locality Preserving Projections (DLPP) [5] encodes discriminant information into LPP to further improve the discriminant performance of LPP for face recognition. These algorithms need to vectorize the objects (samples).

**Figure 1:**
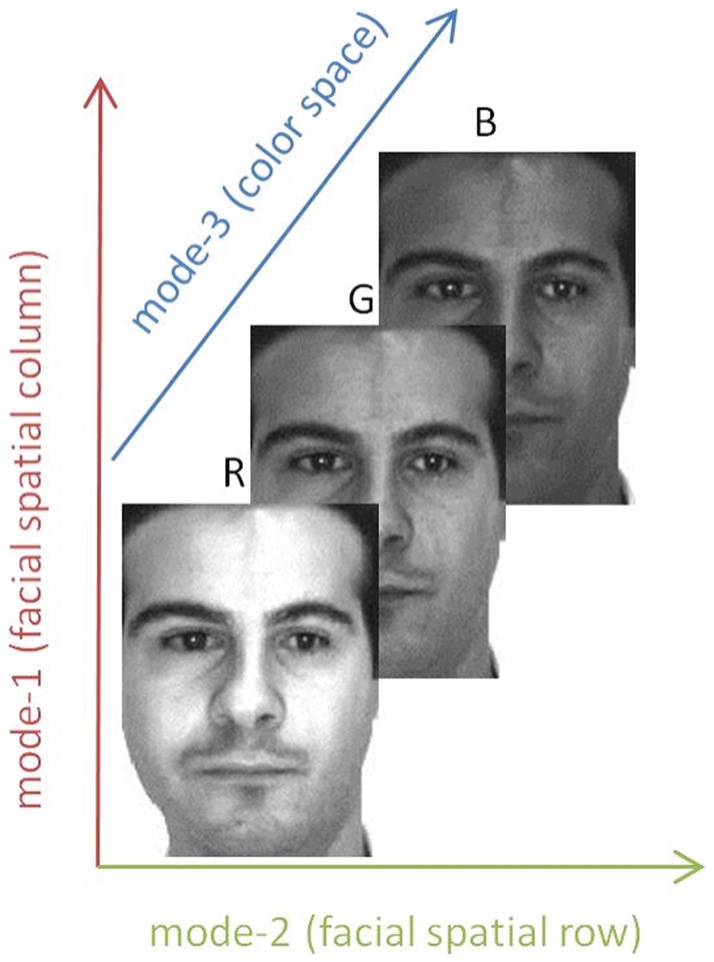
The tensorial represents of a color facial image.

In the real world, however, many objects are naturally represented by multidimensional arrays, i.e., tensors, such as a color facial image used in face recognition (see Fig. 1). If these objects are vectorized, their natural structure information will be lost [6]. As such, a great deal of interests are aroused in the field of tensor [7] [8] [9] [10] [11]. Among the subspace transformation techniques, tensor subspace transformation has also become a highly discussed topic. Multilinear Principal Component Analysis (MPCA) [12], a tensor version of PCA, applies PCA transformation on each mode (or dimensionality) of tensors. Similarly, Discriminant Analysis with Tensor Representation (DATER) [13], General Tensor Discriminant Analysis (GTDA) [14], Tensor Subspace Analysis (TSA) [15], and Discriminant Tensor Subspace Analysis (DTSA) [16] apply LDA, Maximum Scatter Difference (MSD) [17], LPP, and DLPP to transform each mode of tensors, respectively. These tensor subspace transformation methods use a certain vector subspace transformation method to transform every modes of tensors.

However, each mode of tensors may express a different semantic meaning. For example, a color facial image can be treated as a 3rd-order tensor, where mode-1 and mode-2 represent the facial spatial information and mode-3 representing the color space information (see Fig. 1). The facial spatial information and color space information are two different types of information, which should be handled by two different transformations to obtain better performance. In other words, for color facial images, we should implement a transformation strategy on the first two modes and another transformation strategy on the third mode. As such, each type of information should implement a transformation strategy best suited for the semantic meaning.

To the best of our knowledge, there are no existing tensor subspace transformation algorithm, which implements different transformation strategies on different modes of tensors according to their semantic meanings. To address this problem, we propose the fusion tensor subspace transformation framework, which shows an novel idea that different transformation strategies can be implemented on different modes of tensors. Under the framework, we propose the Fusion Tensor Color Space (FTCS) model for face recognition.

## Materials and Methods

### Tensor Fundamentals and Denotations

A tensor is a multidimensional array. It is the higher-order generalization of scaler (zero-order tensor), vector (1st-order tensor), and matrix (2nd-order tensor). In this paper, lowercase italic letters (*a, b,...*) denote scalars, bold lowercase letters (**a, b,...**) denote vectors, bold uppercase letters (**A, B,...**) denote matrices, and calligraphic uppercase letters (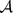, 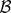,...) denote tensors. The formal definition is given below[18]:

#### Definition 1

*The order of a tensor* 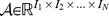 *is* 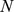 *. An element of* 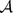 *is denoted by* 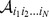 *or* 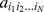 *, where* 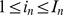 *,* 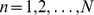


#### Definition 2

*The mode-n vectors of .* 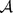 *are the* 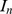 *-dimensional vectors obtained from* 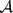 *by fixing every index but index* 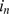


#### Definition 3

*The mode-n unfolding matrix of* 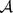 *, denoted by* 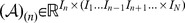 *, contains the element* 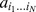 *at* 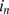 *th row and at* 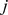 *th column, where*

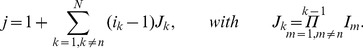
(1)


We can generalize the product of two matrices to the product of a tensor and a matrix.

#### Definition 4

*The mode-n product of a tensor* 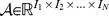 *by a matrix* 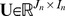 *, denoted by* 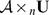 *, is an (* 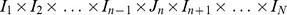 *)-tensor of which the entries are given by:*

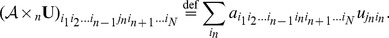
(2)


#### Definition 5

*The scalar product of two tensors* 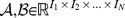 *, denoted by* 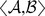 *, is defined in a straightforward way as* 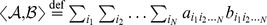 *. The Frobenius norm of a tensor* 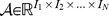 *is then defined as* 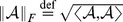


From the definition of the mode-n unfolding matrix, we have
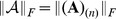
(3)


By using tensor decomposition, any tensor 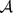 can be expressed as the product
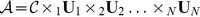
(4)where 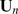, 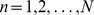, is an orthonormal matrix and contains the ordered principal components for the 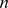
th mode. 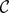 is called the *core tensor*. Unfolding the above equation, we have
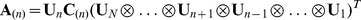
(5)where operator 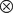 is the Kronecker product of the matrices.

### The Connection among PCA, 2D-PCA and MPCA

Before introducing the fusion tensor subspace transformation framework, we firstly investigate the connection among PCA, 2D-PCA [19] and MPCA. From the previous section, we know that a tensor is the higher-order generalization of scaler, vector and matrix. Similarly, MPCA is the higher-order generalization of PCA and 2D-PCA.

Suppose there are 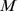 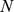-order tensors 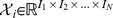, 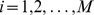. MPCA seeks 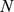 projection matrices 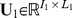, 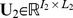,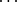, 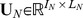 in order to transform 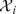 as
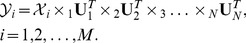
(6)


such that 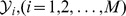 captures most of the variations observed in the original tensor objects 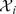.



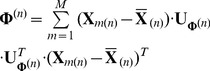
(7)where 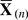 denotes the mode-n unfolding matrix of the mean values of 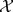, 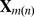 denotes the mode-n unfolding matrix of the mean values of 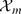 and



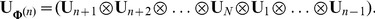
(8)In Eq. (7), 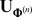 is to use the fixed 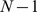 projection matrices 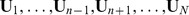 to transform the corresponding 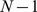 modes.When 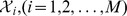 is a 2nd-order tensor, Eq. (6) is simplified to
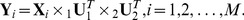
(9)


If we only transform mode-2, 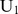, is an identity matrix of size 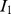. Then, 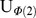 is also an identity matrix and 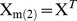. In this case, Eq. (7) is simplified to
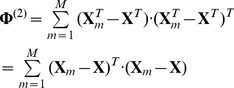
(10)


Eq. (10) is exactly the image covariance (scatter) matrix 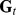 in 2D-PCA [19]. So, 2D-PCA is a special case of MPCA. When objects are represented by matrices and only rows of matrices need to be transformed, MPCA degenerates into 2D-PCA.

When 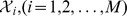 are 1st-order tensors, Eq. (6) is simplified to
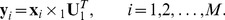
(11)


In this case, Eq (7) is simplified to
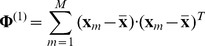
(12)


The above equation is exactly the scatter matrix in PCA. So, PCA is a special case of MPCA. When objects are represented by vectors, MPCA degenerates into PCA.

Following the above analysis, 2D-PCA applies PCA transformation on rows of matrices, and MPCA applies PCA transformation on all modes of tensors.

Similarly, through the above analysis, one can notice that DATER also applies LDA transformation on all modes of tensors. Likewise, GTDA, TSA and DTSA also applies MSD, LPP and DLPP transformation on all modes of tensors respectively. There are several other tensor subspace transformation methods that also applies a single type of transformation on all modes of tensors, however due to page limit we chose to only mention a portion of these algorithms.

### Fusion tensor subspace transformation framework

Tensor subspace transformation method firstly initializes 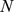 projection matrices 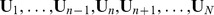 as identity matrices or random matrices, then fixes 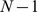 projection matrices 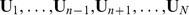. Following, the matrices are used to transform 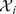, and the transformed results are unfolded on mode-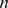. Finally, 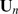 is obtained by implementing a certain transformation on the mode-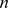 unfolding matrices. We can see that the solution of 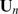 depends on the other projection matrices. 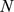 projection matrices are solved by constructing an iterative procedure.

Existing tensor subspace transformation methods only implement one transformation strategy on all modes. In the real world, each mode of tensors may represent a different type of information. We should implement different transformation strategies on different modes according to their semantic meaning. So we propose a Fusion Tensor Subspace Transformation (FTSA) framework, which is described in Table 1. In Table 1, the statement denoted (*) is the core statement in the framework. For a certain represented object, different transformations are used on different modes according to their semantic meaning.

**Table 1:** Fusion tensor subspace transformation framework.

**INPUT**: *M N*-order tensors 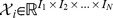 , *i* = 1,2,…,*M*.
**OUTPUT**: U*_n_*, *n* = 1,2,…,*N*
Initialize U*_n_* with a set of identity matrices;
**for** *t* = 1 to *T_max_* **do**
**for** *n* = 1 to *N* **do**
**for** *i* = 1 to *M* **do**
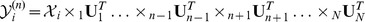 ;
Y*_i(n)_* ← the mode-*n* unfolding matrix of 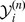 ;
**end for**
*n*-th transformation on *M* matrices Y*_i_*_(*n*)_ to obtain U*_n_*; (*)
**end for**
**if** *t*>2 and 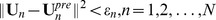 , where 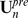 is U*_n_* in the previous iteration.
**break**;
**end if**
**end for**

Under the framework, we developed the Fusion Tensor Color Space (FTCS) model for face recognition.

In the algorithm, we use the maximal iterative times 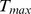 to deal with the problem that the algorithms may be not convergent. Actually, the convergence of many tensor subspace analysis algorithms cannot be generally proved, the classification results based on these algorithms show to be stable after rounds of iterations as illustrated in these papers (e.g. DATER, 2D LDA [20]). The convergence of FTSA depends on the specific transformations.

### Fusion tensor color space model

Recently, researches showed that color information may help to improve the face recognition accuracy. While, the R, G, and B component images in the RGB color space are correlated. Decorrelation among the components of these images helps reduce redundancy and is an important strategy to improve the accuracy of subsequent recognition method [21]. Liu [22] proposed the Uncorrelated Color Space (UCS), the Independent Color Space (ICS), and the Discriminating Color Space (DCS). Specifically, the UCS applies PCA to decorrelate the R, G, and B component images. The ICS and DCS further enhance the discriminating power for the subsequent recognition method by means of Independent Component Analysis (ICA [23]) and LDA, respectively. The experimental results showed that ICS obtains the best color space because its components are not only uncorrelated but also independent.

Many papers have reported that the discriminant analysis methods on facial images can enhance subsequent recognition method [3] [5]. Color Image Discriminant model (CID) [24], borrowing the idea of LDA, aims to seek an optimal color space and an effective recognition method of color images in a unified framework. Tensor Discriminant Color Space (TDCS) [25] model, borrowed the idea of DATER [13], seeks two discriminant projection matrices 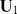, 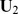 corresponding to the facial spatial information and one color space transformation matrix 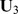 corresponding to the color space. Actually, TDCS uses LDA transformation on both facial spatial information and color space information. They [26] also used elastic net to propose Sparse Tensor Discriminant Color Space (STDCS).

For color space information, however, ICA transformation is better than LDA transformation [22]. Motivated by the insights, we explore a Fusion Tensor Color Space (FTCS) model which applies discriminant analysis on the facial spatial information and applies ICA on the color space information.

A color facial image is naturally represented by a 3rd-order tensor, where mode-1 and mode-2 of a tensor are facial spatial information and mode-3 of tensor is the color space information. For instance, a RGB image with size 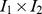 is represented as a tensor 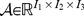, where 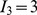. The mode-3 of 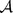 is the color variable in the RGB color space which has 3 components corresponding to 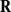, 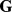 and 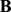 in RGB space. FTCS uses LDA on the first two modes and ICA on the third mode.

Assuming 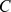 is the number of individuals, 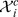 is the 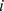
th color facial image of the 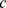
th individual, and 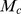 is the number of color facial images of the 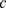
th individual, where 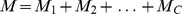. the FTCS algorithm seeks two discriminant projection matrices 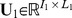, 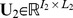 and a color space transformation matrix 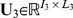 (usually 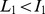, 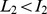 and 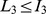) for transformation
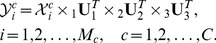
(13)where 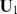 and 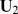 are obtained by using discriminant analysis and 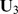 is obtained by using ICA.

The mean image of the 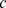-th individual and the mean image of all individuals are defined by:
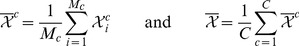
(14)


The between-class scatter and within-class of color images are defined as:
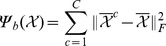
(15)


and
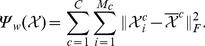
(16)


We can define mode-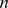 between-class scatter matrix 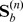 and mode-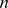 within-class scatter matrix 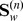 as:
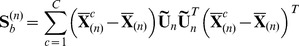
(17)


and
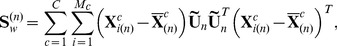
(18)where 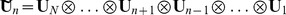, 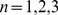.

Then, the between-class scatter of the projected tensors 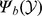 and the within-class scatter of the projected tensors 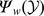 can be rewritten as follows:
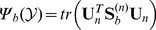
(19)


and
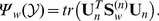
(20)


So, given 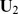 and 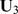 (or 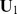,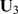), 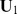 (or 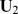) can be obtained by the following discriminant analysis:
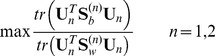
(21)


According to Rayleigh quotient, Eq. (21) is maximized if and only if the matrix 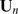 consists of 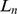 generalized eigenvectors, which corresponds to the largest 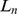 generalized eigenvalues of the matrix pencil 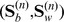, which satisfies:
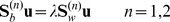
(22)


Since 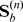 and 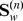 are dependent on 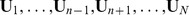, we can see that the optimization of 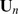 depends on the projections of other modes.

In order to obtain 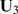, we use ICA (For ICA operations, we used Hyvarinen's fixed-point algorithmhttp://www.cis.hut.fi/projects/ica/fastica/) to decorrelate the RGB color space. we use 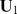 and 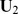, which are obtained through the above discriminant analysis, to transform:
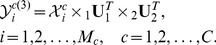
(23)where 
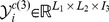
. 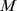 3rd-order tensor 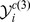 are concatenated to a 4th-order tensor 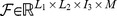. The mode-3 unfolding matrix 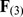 is a 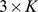 matrix, where 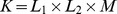 and the three rows of 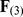 corresponding to the three components in RGB space, respectively.

The color space transformation matrix 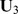 may be derived using ICA on 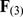. The ICA of 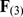 factorizes the covariance matrix 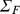 into the following form:
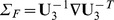
(24)


where 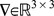 is diagonal real positive and 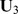 transforms RGB color space to a new color space whose three components are independent or the most independent three component possible. The 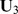 in Eq. (24) may be derived using Comon's ICA algorithm by calculating mutual information and high-order statistics. As a result, an iterative procedure can be constructed to obtain 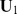, 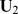 and 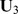.

## Results

### Experiments and results on the AR database

**Figure 2:**
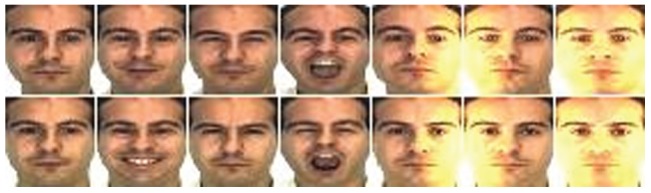
Sample images of one individual from the AR database.

The AR database contains over 4,000 color facial images of 126 people. Each individual participated in two photo sessions. In both sessions, the pictures were taken under identical requirements and conditions. In our experiments, we selected 100 people, where 14 images of each individual are selected and occluded face images are excluded. These facial images have been cropped [27] and can be downloaded from the AR face database official web (http://www2.ece.ohio-state.edu/ aleix/ARdatabase.html). All images are cropped and resized to 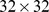 pixels. The sample images for one individual of the AR database are shown in Fig. 2, where the images on the top row are from the first session as the training set, and the images on the bottom row are from the second session as the testing set.

In this experiment, we trained FTCS, TDCS (Although STDCS [26] is better than TDCS, we still did not compare FTCS to STDCS. Because the motivation of the paper is to implement different transformations on different modes of a tensor.) and CID. The convergence threshold 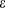 was set as 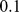 and 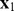 was initialized as 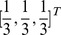. In this case, we got three color space transformation matrices:
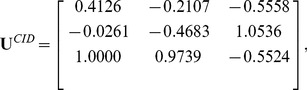
(25)

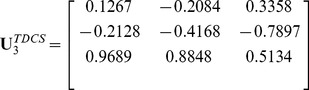
(26)


and
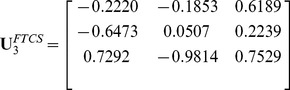
(27)


**Figure 3:**
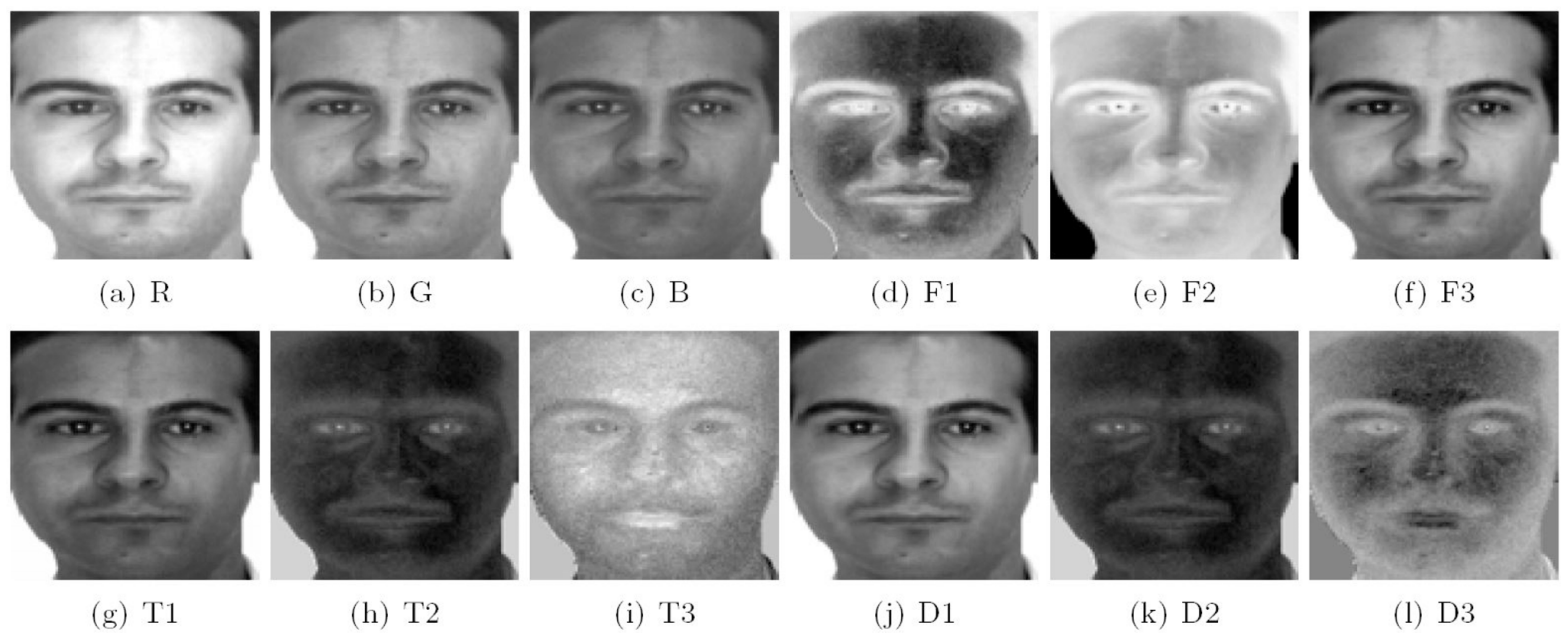
Illustration of R, G, and B color components and the various components generated by CID, TDCS and FTCS on the AR face database.

Using these three matrices, we obtained three color components 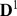, 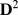, 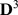 of CID; three color components 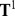, 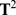, 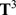 of TDCS and three color components 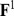, 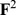, 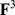 of FTCS (see Fig. 3).

**Figure 4:**
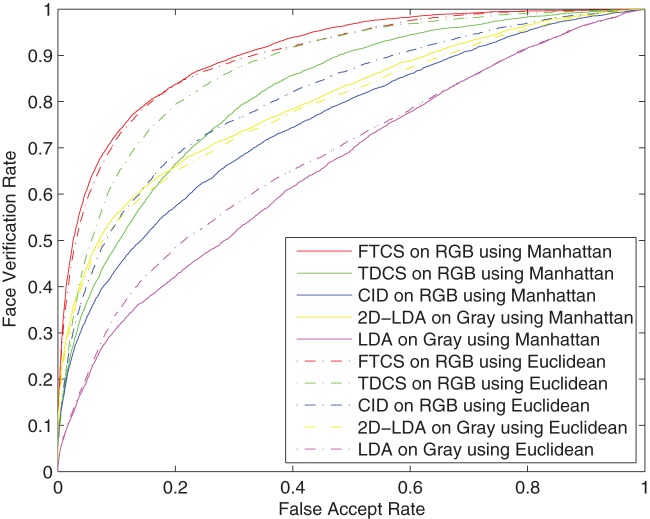
ROC curves of FTCS, TDCS, CID, 2D-LDA and LDA on the AR face database.

Meanwhile, we carried out LDA and 2D-LDA on corresponding gray images. In LDA and CID, only 99 discriminant projection basis vectors were extracted. For 2D-LDA, TDCS and FTCS, the spatial dimensions of the two modes are both reduced to 10. The score matrices were generated by Manhattan distance and Euclidean distance, respectively. The ROC curves of the five methods are shown in Fig. 4. The results indicate that the performance of FTCS with Manhattan distance obtains the best performance. However, the space between two curves of FTCS is narrower than the space between two curves of TDCS. This shows that FTCS is more robust to the type of distance used and results of both Manhattan and Euclidean distance produces closer results than those of TDCS.

In the five algorithms, LDA and CID are used on vectorized face images. Overall, their performances are poorer than the other three algorithms based on tensorized face images. This shows that the facial spatial structure information is important to the face recognition. Specially, when the false accept rate is less than 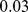, the performance of 2D-LDA outperforms that of TDCS with the color information. In the case, the color information fails to work for face recognition. This is due to the fact that the color space information is transformed by LDA, which is not an optimal transformation for color space information in comparison to ICA. Whereas, FTCS uses ICA on the color space information. As a results, FTCS obtains the best performance.

Table 2 list the verification rates of the five methods with 0.1 FAR. In both cases of Manhattan distance and Euclidean distance, FTCS gets the best verification rate among the five methods. For Manhattan distance, 2D-LDA without the color information is better than TDCS with the color information. Whereas, FTCS outperforms 2D-LDA. This also shows that ICA is better than LDA to transform the color space information.

**Table 2:** Verification rate (in percent) comparison of the five methods, respectively, when the FAR is 0.1.

	FTCS	TDCS	CID	2D-LDA	LDA
Manhattan	71.97	48.10	42.99	54.82	30.29
Euclidean	70.73	62.63	52.70	53.14	33.50

### Experiments and results on the LFW face database



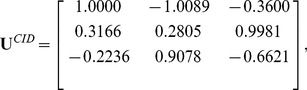
(28)

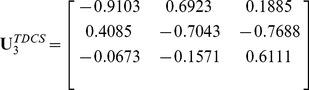
(29)


and
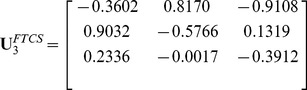
(30)


These components are illustrated in Fig. 6.

**Figure 5:**
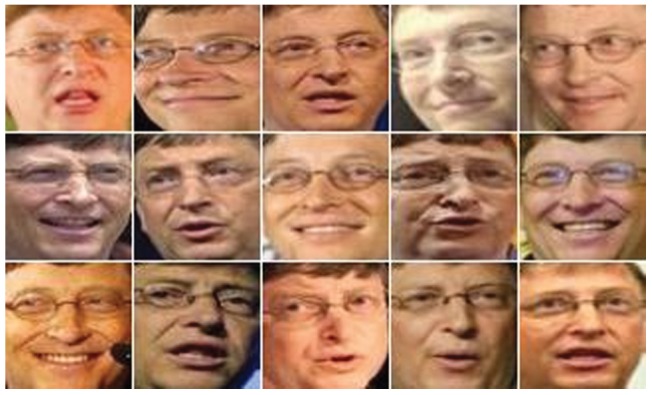
Sample images of one individual from the LFW database.

**Figure 6:**
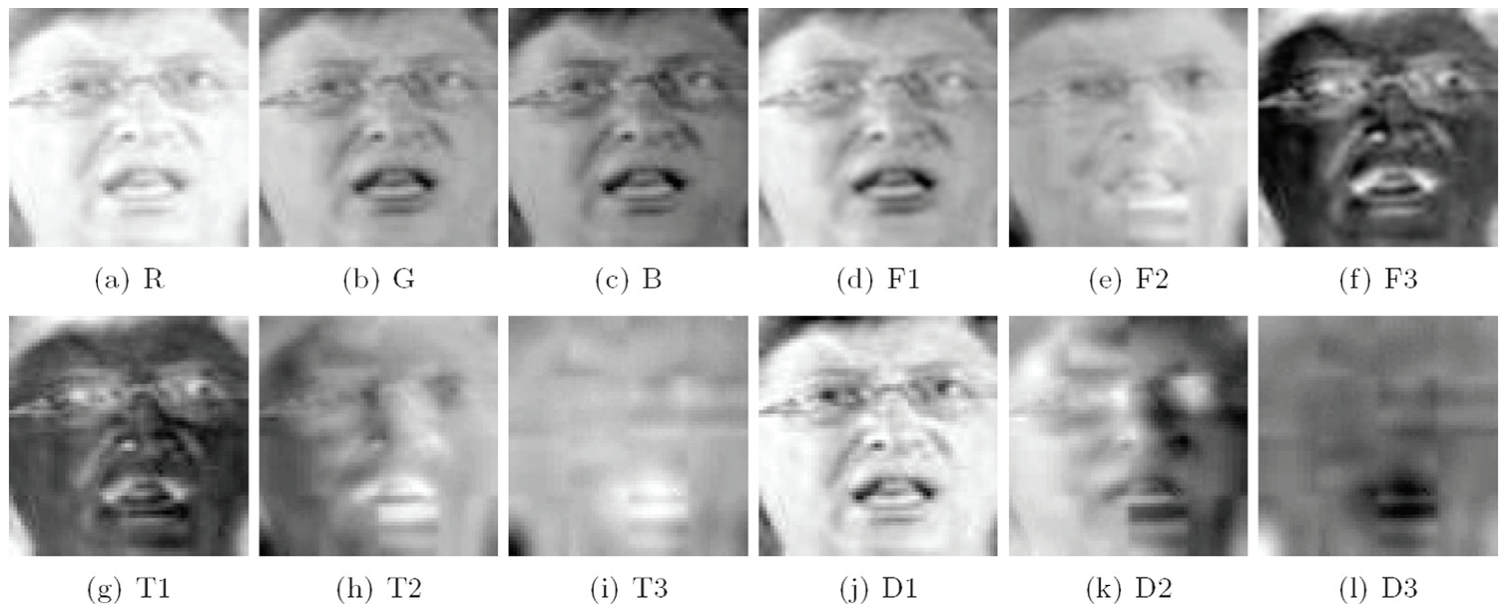
Illustration of R, G, and B color components and the various components generated by CID, TDCS and FTCS on the LFW face database.

**Figure 7:**
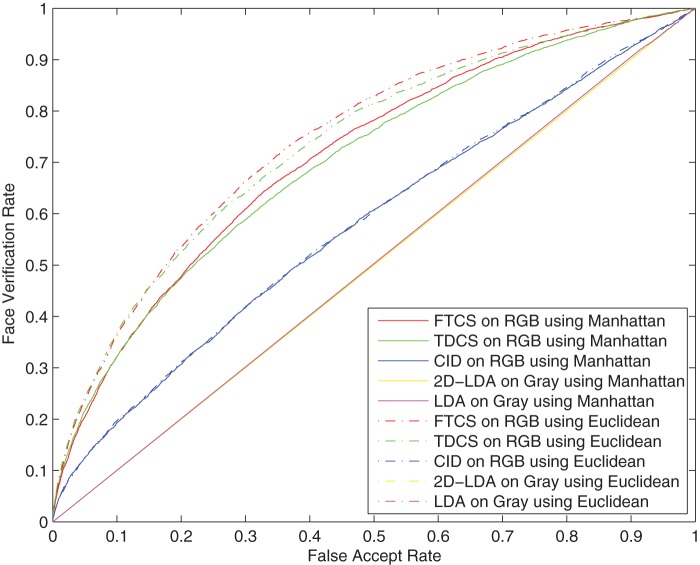
ROC curves of FTCS, TDCS, CID, 2D-LDA and LDA on the LFW face database. Here, 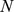 projection matrices 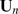 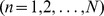 need to be determined. Generally, we fix 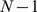 projection matrices 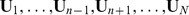 to solve for 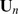. In MPCA, 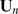 consists of the 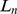 eigenvectors corresponding to the largest 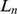 eigenvalues of the matrix

These three matrices are not the same as Eq. (25), Eq. (26) and Eq. (27) due to the different training sets. Using these three matrices, we got three color components 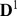, 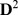, 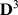 of CID; three color components 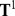, 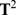, 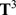 of TDCS and three color components 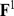, 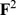, 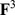 of FTCS.

## Discussion

Recently, tensor subspace transformation is a highly mentioned topic, because many real objects can be represented by tensors. For different objects, the semantic meaning of tensorial modes are different. Even when the objects are the same, each mode of tensors may express a different semantic meaning. To the best of our knowledge, there aren't any existing tensor subspace transformation algorithms which implements different transformation strategies on different mode of tensors according to their semantic meaning. In this paper, we propose the fusion tensor subspace analysis framework, which shows an novel idea that different transformation strategies can applied on different modes of tensor. Under the framework, we propose FTCS for face recognition. The experimental results show the performances of the proposed algorithm is better than existing tensor subspace transformation algorithms. FTCS is only an example of fusion tensor subspace transformation framework. Under the framework, many algorithms can be developed for action recognition, micro-expression recognition, EEG recognition and so on.

## References

[1] Wang X, Tang X (2004) A unified framework for subspace face recognition. IEEE Transactions on Pattern Analysis and Machine Intelligence 26: 1222–1228. 15742896 10.1109/TPAMI.2004.57

[2] Turk M, Pentland A (1991) Eigenfaces for recognition. Journal of Cognitive Neuroscience 3: 71–86. 23964806 10.1162/jocn.1991.3.1.71

[3] Belhumeur PN, Hespanha JP, Kriegman DJ (1997) Eigenfaces vs. Fisherfaces: recognition using class specific linear projection. IEEE Transactions on Pattern Analysis and Machine Intelligence 19: 711–720.

[4] He XF, Niyogi P (2004) Locality preserving projections. Advances In Neural Information Processing Systems 16 16: 153–160.

[5] Yu WW, Teng XL, Liu CQ (2006) Face recognition using discriminant locality preserving projections. Image and Vision Computing 24: 239–248.

[6] Lu H, Plataniotis K, Venetsanopoulos A (2011) A survey of multilinear subspace learning for tensor data. Pattern Recognition 44: 1540–1551.

[7] Tao D, Li X, Wu X, Hu W, Maybank SJ (2007) Supervised tensor learning. Knowledge and Information Systems 13: 1–42.

[8] Zhang L, Zhang L, Tao D, Huang X (2011) A multifeature tensor for remote-sensing target recognition. Geoscience and Remote Sensing Letters, IEEE 8: 374–378.

[9] Tao D, Li X, Wu X, Maybank S (2008) Tensor rank one discriminant analysis – convergent method for discriminative multilinear subspace selection. Neurocomputing 71: 1866–1882.

[10] Tao D, Song M, Li X, Shen J, Sun J, et al (2008) Bayesian tensor approach for 3-d face modeling. Circuits and Systems for Video Technology, IEEE Transactions on 18: 1397–1410.

[11] Sun J, Tao D, Papadimitriou S, Yu PS, Faloutsos C (2008) Incremental tensor analysis: Theory and applications. ACM Transactions on Knowledge Discovery from Data (TKDD) 2: 11.

[12] Lu HP, Konstantinos NP, Venetsanopoulos AN (2008) MPCA: Multilinear principal component analysis of tensor objects. IEEE Transactions on Neural Networks 19: 18–39. 18269936 10.1109/TNN.2007.901277

[13] Yan S, Xu D, Yang Q, Zhang L, Tang X, et al (2005) discriminant analysis with tensor representation. CVPR 2005: 11.

[14] Tao D, Li X, Wu X, Maybank S (2007) General tensor discriminant analysis and gabor features for gait recognition. IEEE Transactions on Pattern Analysis and Machine Intelligence : 1700–1715. 10.1109/TPAMI.2007.1096 17699917

[15] He X, Cai D, Niyogi P (2005) Tensor subspace analysis. In: In Advances in Neural Information Processing Systems 18 (NIPS). MIT Press.

[16] Wang SJ, Zhou CG, Zhang N, Peng XJ, Chen YH, et al (2011) Face recognition using second-order discriminant tensor subspace analysis. Neurocomputing 74: 2142–2156.

[17] Song F, Zhang D, Mei D, Guo Z (2007) A multiple maximum scatter difference discriminant criterion for facial feature extraction. Systems, Man, and Cybernetics, Part B: Cybernetics, IEEE Transactions on 37: 1599–1606. 10.1109/tsmcb.2007.906579 18179076

[18] Kolda TG, Bader BW (2009) Tensor decompositions and applications. Siam Review 51: 455–500.

[19] Yang J, Zhang D, Frangi AF, Yang JY (2004) Two-dimensional PCA: A new approach to appearance-based face representation and recognition. IEEE Transactions on Pattern Analysis and Machine Intelligence 26: 131–137. 15382693 10.1109/tpami.2004.1261097

[20] Ye J, Janardan R, Li Q (2004) Two-dimensional linear discriminant analysis. In: Neural Information Processing Systems. pp. 1569–1576.

[21] Fukunaga K (1990) Introduction to statistical pattern recognition. Academic Pr.

[22] Liu C (2008) Learning the uncorrelated, independent, and discriminating color spaces for face recognition. IEEE Transactions on Information Forensics and Security 3: 213–222.

[23] Comon P (1994) Independent component analysis, a new concept? Signal processing 36: 287–314.

[24] Yang J, Liu C (2008) Color image discriminant models and algorithms for face recognition. IEEE Transactions on Neural Networks 19: 2088–2098. 19054733 10.1109/TNN.2008.2003187

[25] Wang SJ, Yang J, Zhang N, Zhou CG (2011) Tensor discriminant color space for face recognition. IEEE Transactions on Image Processing 20: 2490–2501. 21356616 10.1109/TIP.2011.2121084

[26] Wang SJ, Yang J, Sun MF, Peng XJ, Sun MM, et al (2012) Sparse tensor discriminant color space for face verification. IEEE Transactions on Neural Networks and Learning Systems 23: 876–888. 24806760 10.1109/TNNLS.2012.2191620

[27] Martinez AM, Kak AC (2001) PCA versus LDA. IEEE Transactions on Pattern Analysis and Machine Intelligence 23: 228–233.

